# Study on an Online Detection Method for Ground Water Quality and Instrument Design

**DOI:** 10.3390/s19092153

**Published:** 2019-05-09

**Authors:** Xiushan Wu, Renyuan Tong, Yanjie Wang, Congli Mei, Qing Li

**Affiliations:** 1 School of Electrical Engineering, Zhejiang University of Water Resources and Electric Power, Hangzhou 310018, China; wuxiushan@cjlu.edu.cn (X.W.); clmei@126.com (C.M.); 2 College of Mechanical and Electrical Engineering, China Jiliang University, Hangzhou 310018, China; tongrenyuan@126.com (R.T.); wangyanjiexx@163.com (Y.W.)

**Keywords:** ground water quality, spectrophotometry, online detector, COD, TOC, turbidity

## Abstract

The online measurement of ground water quality, as one important area of water resource protection, can provide real-time measured water quality parameters and send out warning information in a timely manner when the water resource is polluted. Based on ultraviolet (UV) spectrophotometry, a remote online measurement method is proposed and used to measure the ground water quality parameters chemical oxygen demand (COD), total organic carbon (TOC), nitrate nitrogen (NO_3_–N), and turbidity (TURB). The principle of UV spectrophotometry and the data processing method are discussed in detail, the correlated mathematical modeling of COD and TOC is given, and a confirmatory experiment is carried out. Turbidity-compensated mathematical modeling is proposed to improve the COD measurement accuracy and a confirmatory experiment is finished with turbidity that ranges from 0 to 100 NTU (Nephelometric Turbidity Unit). The development of a measurement instrument to detect the ground water COD, TOC, NO_3_–N, and TURB is accomplished; the test experiments are completed according to the standard specification of China’s technical requirement for water quality online automatic monitoring of UV, and the absolute measuring errors of COD, TOC, and NO_3_–N are smaller than 5.0%, while that of TURB is smaller than 5.4%, which meets the requirements for the online measurement of ground water quality.

## 1. Introduction

As an important part of fresh water resources, ground water is an important resource that is required for all living things on Earth to survive and it is also the basic resource for human production and living [1,2,3,4]. Many countries are speeding up the construction of ground water monitoring projects, improving ground water monitoring systems, and establishing national groundwater information management systems to enhance the protection of groundwater resources [5,6]. The online detection methods and design of the detection instruments are important work in the protection of ground water resources [7,8].

The main analysis methods in the field of ground water quality analysis are electrochemical analysis [9,10,11], atomic absorption spectrometry (AAS) [12], gas chromatography [13,14], and spectrophotometry [15,16]. Electrochemical analysis methods have advantages, such as simple operation, fast detection speed, and high precision; however, the service cycle of its electrode is short and the detection process can cause secondary pollution to the environment, which makes this method unsuitable for the field of water quality online monitoring. AAS mainly focuses on the analysis of microelements and it has been widely used in detecting heavy metals in water over recent years. This method requires different light sources to detect different substances, but it is still difficult to use this method for non-metal detection, and this method is therefore not suitable for the field of water quality on-line monitoring. Gas chromatography is a novel detection method, but it is rarely used for online water quality detection at present due to technical limitations. The wavelength is divided into the ultraviolet region, visible region, and infrared region, and the ultraviolet region and visible region for spectrophotometry, the ultraviolet region, and visible region are most commonly used in water quality detection. When compared with other methods, spectrophotometry has such advantages as multiple detection parameters, fast speed, wide range, low analysis cost, and convenient operation. According to the detection parameters that are required for ground water quality, we selected the ultraviolet (UV) region to use as the absorption spectral region of spectrophotometry, that is, we adopted UV spectrophotometry to realize the online detection of ground water quality [17].

A multiple-parameter online monitor of ground water quality is a kind of automatic water quality analyzer specifically for ground water. This instrument realizes the collection and analysis of a water sample, as well as data transmission through remote online operation. This kind of water quality monitor has functions, such as as automatic diagnosis, automatic calibration, automatic failure warning, automatic water sampling, and automatic cleaning. In addition, in the context of ensuring the accurate analysis of detection data, it can realize automatic unattended operation [18,19].

In this study, first, the authors propose a remote online detection method to measure ground water quality parameters that are composed of chemical oxygen demand (COD) [20], total organic carbon (TOC) [21], nitrate nitrogen (NO_3_–N) [22], and turbidity (TURB) [23,24]. Section 2 describes the principle of UV spectrophotometry and the data processing methods in detail. The correlated mathematical modeling of COD and TOC is given, and a confirmatory experiment was carried out. Turbidity-compensated mathematical modeling is proposed to improve the COD measurement accuracy with turbidity ranging from 0 to 100 NTU (Nephelometric Turbidity Unit). Section 3 introduces the multiple-parameter online ground water quality detector design process and structure, which is composed of a detection unit, pipe unit, and control unit. Section 4 presents the test results of the implemented instrument and discussion, followed by the conclusions and future work in Section 5. 

## 2. The Measurement Method and Principle of UV Spectrophotometry 

### 2.1. The Measurement Principle of UV Spectrophotometry

The COD concentration (*C*_COD_) in ground water is determined through the absorbance of ultraviolet light at a wavelength of 254 nm; this is because organic matter has a significant absorption effect on the water parameter measurement at the ultraviolet wavelength of 254 nm. Research and experiments have indicated that there is a certain degree of correlation between COD and TOC in a stable water body, so the concentration of TOC (*C*_TOC_) can be indirectly calculated through a linear correlation with COD [25]. The linear correlation between COD and TOC in a stable water body can be expressed as

(1) $$C_{\mathrm{TOC}}=A\times C_{\mathrm{COD}}+B$$

where the values of *A* and *B* can be obtained by modeling a certain groundwater monitoring point to be measured and the units of *C*_TOC_ and *C*_COD_ are mg/L. 

The principle of using UV spectrophotometry to measure the NO_3_–N concentration is similar to that of *C*_COD_, but two wavelengths of 220 nm and 275 nm are adopted to measure the NO_3_–N content, because the organic matter and nitrate have quite a strong absorption effect at the ultraviolet wavelength of 220 nm, but nitrate does not have an absorption effect at the ultraviolet wavelength of 275 nm [26]. Taking advantage of this feature, the absorbance at 275 nm ($A_{{\mathrm{NO}}_{3}–N,275\mathrm{nm}}$) can be used to compensate the nitrate at 220 nm ($A_{{\mathrm{NO}}_{3}–N,220\mathrm{nm}}$); subsequently, the final absorbance of nitrate ($A_{{\mathrm{NO}}_{3}–N}$) used to calculate the nitrate content can be expressed as

(2) $$A_{{\mathrm{NO}}_{3}–N}=A_{{\mathrm{NO}}_{3}–N,220\mathrm{nm}}-2A_{{\mathrm{NO}}_{3}–N,275\mathrm{nm}}$$

The main methods used to measure turbidity are visual turbidimetry, scattering analysis, and spectrophotometry. Visual turbidimetry means that the observer compares the turbidity of the water sample with that of a standard turbidity solution by visual observation; thus, the results are related to the experience of the observer and they have quite large measurement errors. Scattering analysis detects the turbidity according to the light scattering principle, which can be divided into vertical scattering, forward scattering, and backward scattering based on the receiving angle and direction of scattering lights. Spectrophotometry is a kind of method that is used to measure the turbidity by establishing the relation between the attenuation of incident light and the turbidity based on the Lambert–Beer law, and through measuring the strength of transmission light and incident light, this method is more precise than visual turbidimetry and it is easier than scattering analysis to operate. 

In practical ground water quality detection, suspended substances in the extracted ground water can significantly influence the detection precision of COD, TOC, and NO_3_–N; thus, turbidity compensation analysis is an essential step in the instrument measurement method. According to spectral analysis, it can be obtained that the light absorptions of COD and NO_3_–N are almost zero when the wavelength of ultraviolet light is 350 nm [27], so the system used ultraviolet light with a wavelength of 350 nm as the compensation light and it cooperated with the absorption wavelength of COD to detect the water quality without influence from turbidity. 

### 2.2. Modeling and Verification of Linear Correlation

#### 2.2.1. Modeling and Verification of the Linear Correlation of COD and TOC

**Figure 1:**
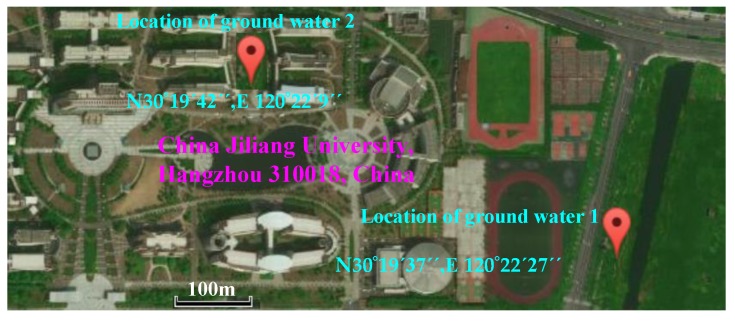
The location of ground water 1 and water 2.

Two ground water samples in Hangzhou were used for experimental modeling in order to verify the linear correlation of COD and TOC, and these were respectively marked ground water 1 and ground water 2, where ground water 1 was close to surface rivers and ground water 2 was far away from surface rivers. Figure 1 shows the water was sampled from China Jiliang University, Jianggan district of Hangzhou, Zhejiang Province, People’s Republic of China.

**Figure 2:**
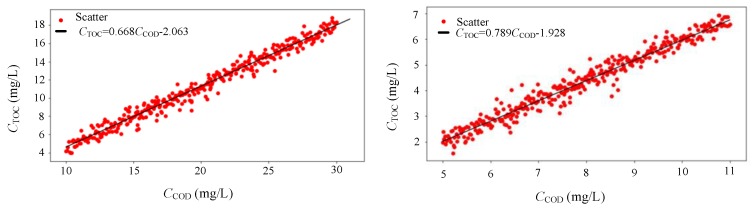
Chemical oxygen demand/total organic carbon (COD/TOC) relationship line of ground water 1 and ground water 2.

In the experiment, the ground water types, marked ground water 1 and ground water 2, were respectively extracted as samples, and the *C*_COD_ and *C*_TOC_ were detected using a COD–4200 analyzer (Shimadzu, Japan) and a TOC-LCSH/CSN analyzer (Shimadzu, Japan), respectively. In order to make the linear model of COD and TOC more persuasive, the experimental period was stretched to six months. Eight days per month (except rainy days) were selected for sampling, detecting, and recording; finally, modeling and recorded data analysis were conducted while using Python data analysis software. A dot graphic with the detected *C*_COD_ as the *x* axis and *C*_TOC_ as *y* was generated, and then the curvilinear figures that are shown in Figure 2 were obtained by data fitting with the least squares algorithm for ground water 1 and ground water 2, respectively [28]. The linear regression equations of COD and TOC are given in the Figure 2, the goodness of fits are 0.9538 and 0.8858 for the water 1 and water 2, respectively. It can be seen that there was a good linear correlation between COD and TOC at the water sampling locations; in addition, the correlation was stronger at ground water 1. Therefore, for different ground water areas, the indirect determination of the TOC value based on UV spectrophotometry could be realized when the coefficients in equation 1 are obtained by establishing the correlation model of COD and TOC in the ground water areas and obtaining the linear regression equations.

#### 2.2.2. Modeling and Verification of the Relation between COD Absorbance and Turbidity

**Figure 3:**
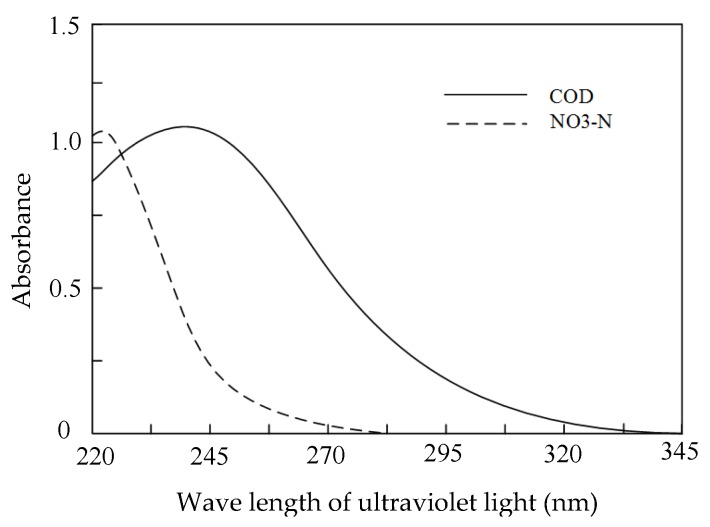
The ultraviolet absorbance of COD and nitrate nitrogen (NO_3_–N).

At present, 640 nm wavelength visible light being used as the turbidity compensation light is a commonly accepted method in water quality detection. For the COD and NO_3_–N parameters in ground water, 350 nm ultraviolet light can be also used as a turbidity compensation light instead of visible light. Figure 3 shoed the absorption spectra of COD and NO_3_–N standard solution in the ultraviolet spectral region [29]. *C*_TOC_ is indirectly obtained from *C*_COD_, so it is not discussed here. It can be seen that the absorbance of COD and NO_3_–N is almost zero when the wavelength of ultraviolet light is 350 nm, so ultraviolet light with a wavelength of 350 nm was used as the compensation light in the instrument system.

**Figure 4:**
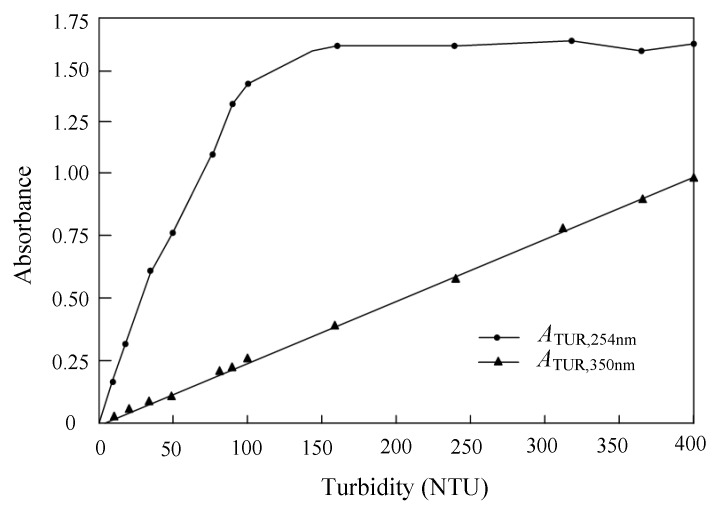
Absorbance curves of turbidity (TURB) at 254 nm and 350 nm.

A Formazin standard turbidity solution of 4000 NTU was diluted into standard turbidity solutions that ranged from 10 to 400 NTU with a gradient of 10 NTU using distilled water; the absorbances of selected different turbidity solutions were measured at the wavelengths of 254 nm and 350 nm using a UV754N visible spectrophotometer (Shanghai Yidian Scientific Instrument Co., Ltd., Shanghai, China) [30]. Figure 4 shows the measured absorbances at the wavelengths of 254 and 350 nm with the solution turbidity. It can be seen that the absorbance at a 254 nm wavelength has a good linear correlation, when the turbidity is from 0 to 100 NTU, but the absorbance at a 350 nm wavelength always remains as a good linear correlation in the range from 0 to 400 NTU; the reason for this characteristic is that the range of absorbance should be controlled within a reasonable section to analyze the relationship between absorbance and concentration based on the Lambert–Beer law. It can be seen that there is a good linear correlation between the absorbance at 254 nm (*A*_TUR,254nm_) and the absorbance at 350 nm (*A*_TUR,350nm_) in the range from 0 to 100NTU, and the linear correlation equation was obtained as

(3) $$A_{\mathrm{TUR},254\mathrm{nm}}=6.1132A_{\mathrm{TUR},350\mathrm{nm}}-0.0289.$$

Based on above analysis, when COD in the solution with low turbidity was measured, the *A*_TUR,350nm_ was firstly measured and transferred to the *A*_TUR,254nm_ through Equation (3), the calculated *A*_TUR,254nm_ was used as the absorbance compensated amount. Finally, the absorbance of low turbidity solution the solution at the 254 nm wavelength for COD is the difference between the measured absorbance and the *A*_TUR,254nm_.

**Figure 5:**
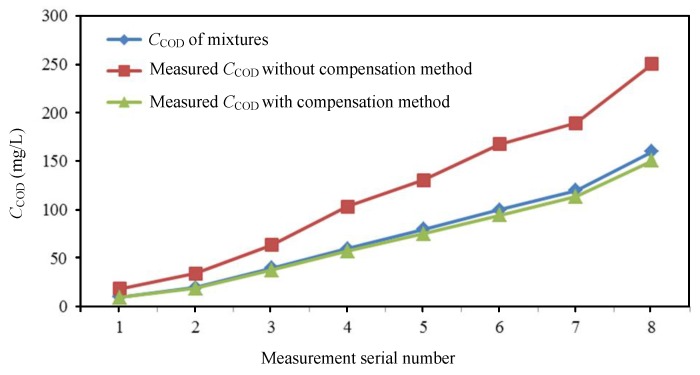
Mixed solution *C*_COD_ measured results.

Two experiments were carried out to detect *C*_COD_ in order to verify the turbidity compensation method’s effectiveness; one was carried out with turbidity compensation and the other without the turbidity compensation. Multiple mixtures with different turbidity and COD concentration were produced in 50 mL quantities; the experimental concentrations of the mixtures were (4,10), (8,20), (16,40), (24,60), (32,80), (40,100), and (48,120), where the first value is *C*_TOC_ and the second value indicates the *C*_COD_. All of the mixtures were divided into two groups; one group adopted the turbidity compensation to detect the *C*_COD_ directly with the ultraviolet light at 254 nm wavelength, and the other one adopted the turbidity compensation to detect the COD concentration. Table 1 and in Figure 5 detail the measured results. The relative error is defined as the percentage of the absolute error divided by the true value. It can be seen that the relative errors after the compensation were much smaller than those without compensation, which proved that the turbidity compensation method could effectively eliminate the COD detection error that is caused by turbidity and also proved the feasibility of a turbidity compensation model that was established at a 350 nm wavelength.

## 3. Apparatus Design

### 3.1. System Structure

**Figure 6:**
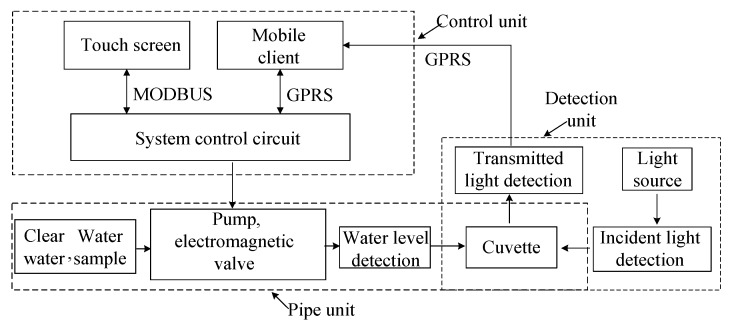
Instrument system block diagram.

The system structure of the designed online ground water quality multiple parameter detector, as shown in Figure 6, is composed of a detection unit, pipe unit, and control unit. The MODBUS-RTU (ModBus Remote Terminal Unit) protocol and GPRS (General Packet Radio Service) protocol were adopted for the remote control requirement.

### 3.2. Detection Unit

**Figure 7:**
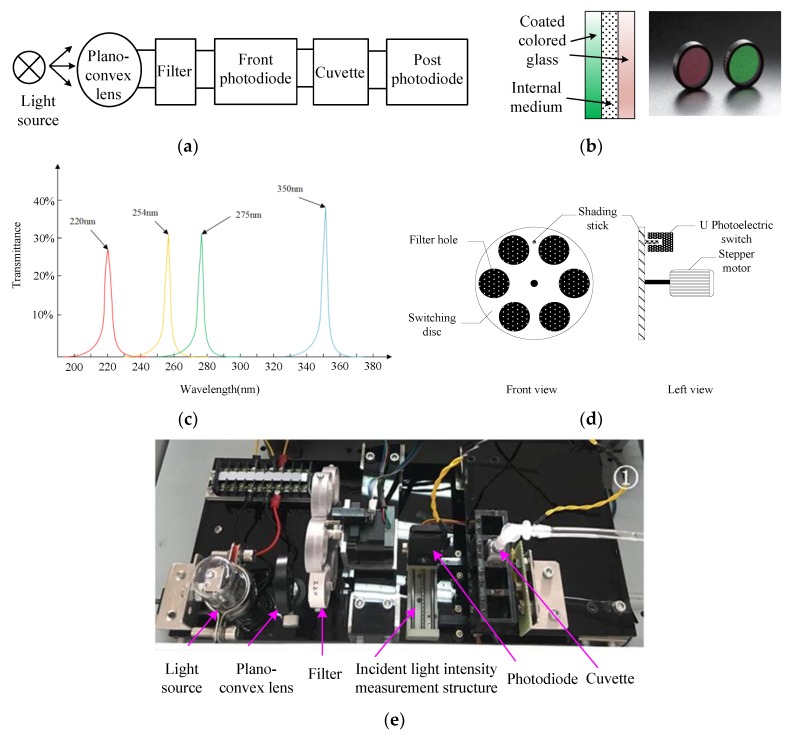
Optical detector unit (**a**) block diagram; (**b**) photograph; (**c**) Filter; (**d**) Filter transmission spectrum; and, (**e**) Automatic switching filter structure diagram.

The design of the detection unit, as shown in Figure 7a, is composed of a light source, quartz plano-convex lens, filter lens, front photodiode, quartz cuvette, and post photodiode. The light source, quartz plano-convex lens, and filter lens are used to produce a single-wavelength parallel light; the front photodiode is used to detect the incident light intensity; and, the post photodiode is used to detect transmitted light intensity. The quartz cuvette between the two photodiodes is used to contain the solution that is to be tested. For the optical detector unit, a TU1810 deuterium lamp (Beijing Purkinie General Instrument Company, Beijing, China) was applied as the light source [31,32]. The filter is composed of two colored glass sheets with an inner surface coating and inner medium sandwiched between the two glass sheets. Figure 7b shows a diagram of the filter. The compound light passes through the filter and then a single-wavelength light can be obtained. Bandpass filters with wavelengths of 220 nm, 254 nm, 275 nm, and 350 nm were selected for the optical detector unit [33], and Figure 7 shows the transmitted light spectrum. The designed automatic switching filter mechanism that is shown in Figure 7c implemented the switching method of the filter. It is composed of a switching disc, U-photoelectric switch, and 12 V stepping motor. Figure 7e shows the completed optical detector unit.

**Figure 8:**
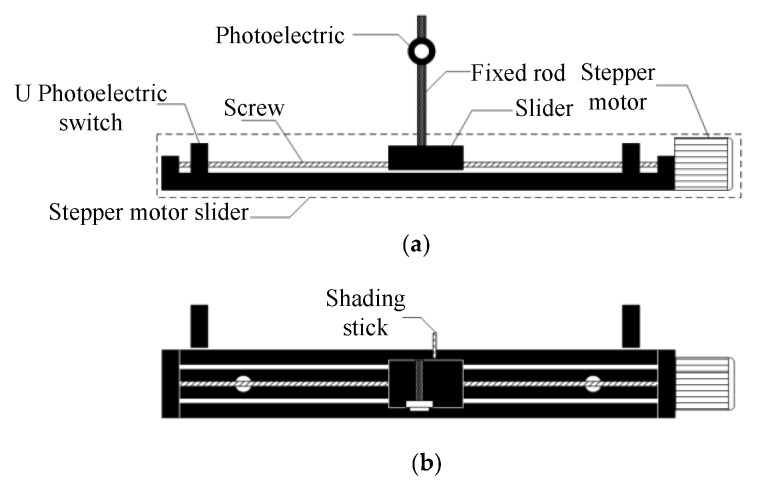
Incident light intensity detection structure diagram: (**a**) front view; and, (**b**) top view.

It is necessary to detect the incident intensity and the transmitted intensity based on the UV spectrophotometry that is used to measure the water quality parameters. It is easier to detect the transmitted intensity when the photodiode is directly installed behind the quartz cuvette, but, for detecting the transmitted intensity, the influence of the optical detector unit on the transmitted intensity should be considered. The light path of the transmitted light is affected by the structure if the photodiode is directly located in front of the quartz cuvette. Therefore, an incident intensity detection mechanism was designed to achieve accurate measurement of the incident intensity. The photodiode is moved into the light path for detecting the incident intensity and it is then moved out of the light path after finishing the detection; this avoids the influence of the photodiode on the incident intensity. The detecting mechanism of incident intensity, as shown in Figure 8, is mainly composed of a slider, screw, stepper motor, photodiode, and U-shape photoelectric switch. The sliding rail screw is rotated by the stepper motor to drive the sliding block and then drive the photodiode on the sliding block. The photodiode is installed at the fixed rod and its position can be adjusted by sliding up or down to ensure that the photodiode and the light path are at the same level. A U photoelectric switch was added at the end of the slider and a shading stick was equipped to the front side of the slider in order to make sure that the photodiode can pass through the optical path when detecting the incident intensity. The shading stick triggers the U photoelectric switch to make sure that the slider can slide back and forth once, then to make sure that the photodiode should pass through the optical path once.

### 3.3. Pipeline Unit

**Figure 9:**
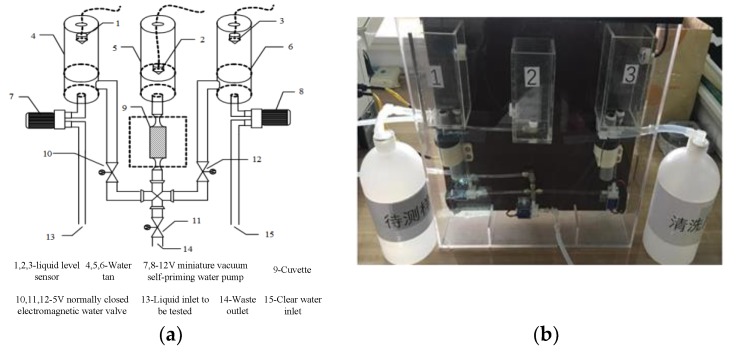
Pipe unit: (**a**) structure diagram; and (**b**) pipe unit photograph.

With the development of water quality detectors, detectors with automatic water sampling and pipe cleaning have been widely sought. Thus, we designed the pipe unit with functions of automatic water sampling and pipe cleaning. The pipe unit structure that is shown in Figure 9a is composed of liquid level sensors, a water tank, a 12 V miniature vacuum self-priming water pump, a quartz cuvette, a 5 V normally-closed electromagnetic water valve, an inlet for liquid to be tested, a waste outlet, and a clear water inlet. The operating modes of the pipe unit are extracting the solution to be tested and clearing the pipe; Figure 9b shows the finished pipe unit. 

### 3.4. System Control Unit

**Figure 10:**
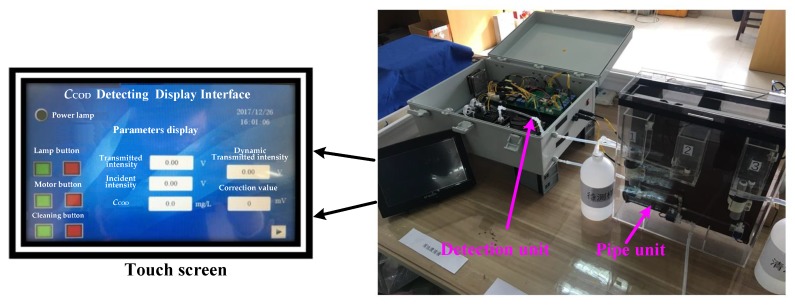
Instrument photograph.

The system control unit is composed of a power circuit, control and detection circuit, communication circuit, and a minimum control system that is based on an STM32 microcomputer. The control and detection circuit consists of a liquid level detection module, electromagnetic valve and water pump control module, motor control module, and light intensity detection module, while the communication circuit is composed of an RS232 serial port communication module and a GPRS transmission module; Figure 10 shows the finished instrument. The software design of the instrument includes the STM32 system control program, communication program, and the interface program of human–computer interaction via touch screen. The detection interface shown in Figure 10 is divided into the key area and the parameter display area.

## 4. Experiment

The four ground water quality parameters COD, NO_3_–N, TURB, and TOC were detected by the implemented instrument based on the specifications of “Technical Requirements for Automatic Monitoring of Ultraviolet (UV) Absorption Water Quality”, as implemented by the Environmental Protection Department of the People’s Republic of China on 11 November 2005. The detection solutions according to the China National Standards were a 1000 mg/L COD standard solution, a 1000 mg/L TOC standard solution, a 1000 mg/L NO_3_–N standard solution, and a 4000 mg/L turbidity standard solution. Table 2 shows the parameters of the standard solutions.

### 4.1. COD Detection Experiment

We diluted the 1000 mg/L COD standard solution with distilled water to 10 different concentrations of COD standard test solutions. The wavelength of the instrument was adjusted to 254 nm and the prepared 10 different concentrations of COD standard test solutions were detected; Table 3 lists the measured results. According to the measured results in the table, a linear fitting method was adopted to analyze the measured results; the curve equation can be expressed as

(4) $$A_{\mathrm{COD},254\mathrm{nm}}=0.005023C_{\mathrm{COD}\_T}+0.01091$$

where *A*_COD,254nm_ is the absorbance and *C*_COD_T_ is the concentration of the COD standard solution. It can be seen that there is a good linear correlation between COD and the absorbance in a certain concentration range [34]. We can detect the COD standard solutions with different concentrations to avoid the contingency of indication error. We detected the COD standard solution of the same concentration four times, and Table 4 lists the detected results. The maximum measured error was less than 4.79% in the concentration range from 0 to 500 mg/L.

### 4.2. TOC Detection Experiment 

The measurement method for the TOC detection was similar to that for COD detection. The TOC standard solution was diluted to 10 different concentrations of TOC standard test solutions. The wavelength of the instrument was adjusted to 254 nm and the diluted 10 different concentrations of TOC standard test solutions were detected; Table 5 lists the measured absorbances.

According to the measured results in Table 5, a linear fitting method was adopted to analyze the measured results; and the curve equation can be expressed as

(5) $$A_{\mathrm{TOC},254\mathrm{nm}}=0.01745C_{\mathrm{TOC}\_T}+0.0060346$$

where *A*_TOC,254nm_ is the absorbance and *C*_TOC_T_ is the concentration of the TOC standard solution. It can be seen that there is a good linear correlation between TOC and the absorbance for concentrations, which range from 0 to 100 mg/L. We detected the TOC standard solution of the same concentration 4 times to avoid the contingency of indication error. The detected results are listed in Table 6 and the maximum measured error was less than 4.89% in the concentration range from 10 to 100 mg/L.

### 4.3. NO_3_–N Detection Experiment

The NO_3_–N standard solution was diluted to 10 different concentrations of NO_3_–N standard test solutions. According to the detection method for NO_3_–N, ultraviolet light with wavelengths of 220 nm and 275 nm were used to detect the different concentrations of the NO_3_–N standard solutions [35]. Table 7 lists the measured absorbances at 220 nm (*A*_NO3__–__N,220nm_) and 275 nm (*A*_NO3__–__N,275nm_). Based on the experimental results, the fitted curve equation that was calculated by the absorbance correction formula is given by

(6) $$A_{{\mathrm{NO}}_{3}–N,220\mathrm{nm}}=0.013519C_{{\mathrm{NO}}_{3}–N\_T}+0.00112648$$

where *C*_NO__3__–__N___T_ is the concentration of the NO_3_–N standard solution. We detected the NO_3_–N standard solution of the same concentration four times to avoid the contingency of indication error. Table 8 lists the detected results, and the maximum measured error was less than 4.83% in the concentration range from 1.0 to 10.0 mg/L.

### 4.4. TURB Detection Experiment

The Formazin standard solution was diluted to 10 different concentrations of turbidity standard test solutions. According to the detection method for turbidity, ultraviolet light with a wavelength of 350 nm was used to detect the different concentrations of turbidity standard solutions. Table 9 lists the measured absorbance (*A*_TUR,350nm_) values, and, based on the experimental results, the fitted curve equation was obtained as

(7) $$A_{\mathrm{TUR},350\mathrm{nm}}=0.0022C_{\mathrm{TUR}\_T}+0.0157$$

where *C*_TUR_T_ is the concentration of the turbidity standard solution. We detected the turbidity standard solution of the same concentration four times to avoid the contingency of indication error. Table 10 lists the detected results, and the maximum measured error was less than 5.84% in the concentration range from 0 to 400 NTU.

## 5. Conclusions

In this paper, based on UV spectrophotometry, a remote online measurement method was proposed and used to measure the ground water quality parameters COD, TOC, NO_3_–N, and TURB. The principle of UV spectrophotometry and the data processing method were discussed in detail. The following conclusions were drawn based on the theoretical analysis and experimental results.

(1) The ground water quality parameters COD, TOC, NO_3_–N, and TURB were detected by the designed instrument and the detected relative errors were smaller than 5.0%, which proves that the proposed method of measuring multiple parameters of ground water quality based on UV spectrophotometry is feasible.
(2) There is a certain correlation between COD and TOC in a stable water body. TOC content can therefore be obtained indirectly from detected COD through the linear correlation to COD; the experiment verified this.
(3) The suspended substances in ground water have significant influences on the detection of COD, TOC, and NO_3_–N, so carrying out turbidity compensation analysis of the system when the COD, TOC, and NO_3_–N are detected is an essential step. For detecting the COD concentration of the ground water, the absorbance at the wavelength of 350 nm could be measured in advance and transferred to the absorbance at the wavelength of 254 nm based on Equation (3).
(4) The wavelengths of 220 nm and 275 nm were used to measure the NO_3_–N concentration, because both organic matter and NO_3_–N have a strong absorption effect at 220 nm of ultraviolet light, but NO_3_–N does not have an absorption effect at 275 nm.

Our future work will focus on the study of NO_3_–N turbidity compensation. In the process of water quality detection, except for suspended substances, there is also other water-soluble matter, which may affect the detection, so we shall reduce the impact of these matters on the detection, so as to further improve the detection precision of the instrument.

## Figures and Tables

**Table 1:** Mixed solution COD concentration measured results and relative errors.

Serial Number	Mixed Solution *C*_COD_ (mg/L)	Measured *C*_COD_ without Compensation (mg/L)	Relative Error without Compensation (%)	Measured *C*_COD_ with Compensation (mg/L)	Relative Error with Compensation (%)
1	10	18.56	85.6	9.57	−4.30
2	20	34.12	70.6	18.98	−5.10
3	40	63.67	59.2	37.90	−5.25
4	60	103.24	72.1	57.49	−4.10
5	80	130.50	63.1	75.39	−5.80
6	100	167.83	67.8	94.56	−5.44
7	120	189.34	57.8	113.66	−5.20
8	160	250.59	56.6	150.78	−5.76

**Table 2:** Standard solutions for testing the instrument.

Standard Solution Category	China National Standard Sample Number	Identification	Ingredients	Medium	Relative Expansion Uncertainty
COD	GBW (E) 081786	174960-3	C_8_H_5_KO_4_	H_2_O	*U* = 1% *K* = 2
TOC	GSB 07-1967-2005	174860-3	C_8_H_5_KO_4_	H_2_O	*U* = 2% *K* = 2
NO_3_–N	GSB 04-1772-2004	175059-3	NO_3-_	H_2_O	*U* = 2% *K* = 2
TURB	SGB-YQT01028H	174760-3	Formazine	H_2_O	*U* = 2% *K* = 2

**Table 3:** Absorbance of different concentrations of COD standard solution at 254 nm.

*C*_COD_T_ (mg/L)	*A*_COD,254nm_	*C*_COD_T_ (mg/L)	*A*_COD,254nm_
50.00	0.262	300.00	1.581
100.00	0.513	350.00	1.769
150.00	0.764	400.00	2.201
200.00	1.015	450.00	2.271
250.00	1.267	500.00	2.522

**Table 4:** Measured results and relative errors for different concentrations of COD solution.

*C*_COD_T_ (mg/L)	Measured Results (mg/L)				Average Value (mg/L)	Relative Error (%)
	No.1	No.2	No.3	No.4		
50.00	52.33	52.23	51.49	53.46	52.37	4.75
100.00	102.11	102.23	103.59	104.99	103.23	3.23
150.00	154.11	155.67	155.34	159.45	156.14	4.10
200.00	205.34	201.33	203.45	204.55	203.66	1.83
250.00	248.44	246.78	245.99	247.55	247.69	−1.12
300.00	311.20	309.19	310.42	315.33	311.53	3.85
350.00	348.45	347.66	345.89	346.01	347.00	−0.86
400.00	410.45	411.4	412.44	412.34	411.65	2.91
450.00	456.56	455.59	457.88	459.79	457.45	1.65
500.00	510.00	528.89	529.89	529.98	530.98	4.79

**Table 5:** Absorbance of different concentrations of TOC standard solution at 254 nm.

*C*_TOC_T_ (mg/L)	*A*_TOC,254nm_	*C*_TOC_T_ (mg/L)	*A*_TOC,254nm_
10.00	0.181	60.00	1.153
20.00	0.353	70.00	1.228
30.00	0.480	80.00	1.403
40.00	0.714	90.00	1.577
50.00	0.879	100.00	1.752

**Table 6:** Measured results and relative errors for different concentrations of TOC solution.

*C*_TOC_T_ (mg/L)	Measured Results (mg/L)				Average Value (mg/L)	Relative Error (%)
	No.1	No.2	No.3	No.4		
10.00	10.58	10.39	10.43	10.35	10.44	4.37
20.00	21.10	21.05	20.89	20.99	21.00	4.89
30.00	29.79	29.90	30.02	29.87	29.89	−3.50
40.00	41.89	42.05	41.78	41.86	41.90	4.73
50.00	52.90	52.49	52.06	51.99	52.36	4.72
60.00	58.04	57.88	57.89	57.90	57.93	−3.45
70.00	67.30	67.50	67.34	67.20	67.34	−3.81
80.00	83.80	83.90	84.10	84.10	84.98	4.97
90.00	89.00	88.70	88.67	88.60	88.74	−1.40
100.00	104.90	104.48	103.59	104.10	104.27	4.27

**Table 7:** Absorbance of different concentrations of NO_3_–N standard solution at 220 nm and 275nm.

*C*_NO3–N_T_ (mg/L)	*A*_NO3–N,220nm_	*A*_NO3–N,275nm_	*C*_NO3–N_T_ (mg/L)	*A*_NO3–N,220nm_	*A*_NO3–N,275nm_
1.00	0.017	0.001	6.00	0.085	0.001
2.00	0.028	0.000	7.00	0.098	0.001
3.00	0.044	0.001	8.00	0.110	0.000
4.00	0.055	0.000	9.00	0.123	0.000
5.00	0.070	0.000	10.00	0.140	0.002

**Table 8:** Measured results and relative errors for different concentrations of NO_3_–N solution.

C_NO3–N_T_ (mg/L)	Measured Results (mg/L)				Average Value (mg/L)	Relative Error (%)
	No.1	No.2	No.3	No.4		
1.00	0.95	0.98	0.97	0.98	0.97	−3.00
2.00	1.93	1.95	1.95	1.94	1.94	−2.88
3.00	3.08	3.08	3.06	3.05	3.07	2.25
4.00	4.05	4.05	4.06	4.05	4.05	1.31
5.00	4.92	4.95	4.96	4.95	4.94	−1.10
6.00	5.92	5.95	5.90	5.93	5.93	−1.25
7.00	7.20	7.15	7.10	7.11	7.14	2.00
8.00	8.25	8.27	8.25	8.30	8.27	3.34
9.00	8.70	8.73	8.69	8.65	8.69	−3.42
10.00	10.50	10.48	10.45	10.50	10.48	4.83

**Table 9:** Absorbance of different concentrations of TURB standard solution at 350 nm.

*C*_TUR_T_ (mg/L)	*A*_TUR,350nm_	*C*_TUR_T_ (mg/L)	*A*_TUR,350nm_
40.00	0.103	240.00	0.573
80.00	0.192	280.00	0.632
120.00	0.280	320.00	0.720
160.00	0.388	360.00	0.808
200.00	0.496	400.00	0.894

**Table 10:** Measured results and relative errors for different concentrations of TURB solution.

*C*_TUR_T_ (NTU)	Measured Results (NTU)				Average Value (NTU)	Relative Error (%)
	No.1	No.2	No.3	No.4		
40.00	41.10	42.50	42.20	42.54	42.09	5.21
80.00	84.15	85.59	85.23	82.34	84.32	5.40
120.00	115.49	116.67	113.99	112.32	114.62	−4.49
160.00	158.90	155.23	156.29	157.89	157.08	−1.83
200.00	205.23	207.84	206.34	204.79	206.05	3.03
240.00	245.46	243.26	244.67	244.54	244.48	1.87
280.00	275.45	278.33	277.99	276.80	277.14	−1.02
320.00	310.40	312.55	316.78	313.20	313.23	2.16
360.00	380.60	381.30	382.52	379.68	381.03	5.84
400.00	407.46	408.26	407.47	405.54	405.98	1.80
